# A FEASIBILITY STUDY OF DIGITAL SELF-REPORT MEASUREMENT FOR BRAIN INJURY PATIENTS UTILIZING AN ADAPTED VERSION OF THE MAYO-PORTLAND ADAPTABILITY INVENTORY – FOURTH EDITION

**DOI:** 10.2340/jrm.v57.43644

**Published:** 2025-10-07

**Authors:** Mikael GEWERS, Kristian BORG, Uno FORS, Sabine KOCH, Marika C. MÖLLER, Aniko BARTFAI

**Affiliations:** 1 Department of Clinical Sciences, Danderyd Hospital, Karolinska Institutet, Stockholm; 2 Department of Rehabilitation Medicine, Danderyd University Hospital, Stockholm; 3 Department of Computer and Systems Sciences, Stockholm University, Stockholm; 4 Department of Learning, Informatics, Management and Ethics, Health Informatics Centre, Karolinska Institutet, Stockholm, Sweden

**Keywords:** brain injuries, nervous system diseases, self-assessment, eHealth

## Abstract

**Objectives:**

This study aimed to examine the clinical relevance and usability of the digital self-report version of the Mayo-Portland Adaptability Inventory – fourth edition, MPAI-4 (MPAI-4-S-dig). In its paper version, MPAI-4 is well validated for patients with acquired brain injuries (ABIs) and neurological disorders (NDs), but time consuming. An additional aim was to investigate whether MPAI-4-S-dig is reliable for repeated measurements.

**Setting:**

Community neurorehabilitation in Stockholm, Sweden.

**Methods:**

MPAI-4-S-dig was administered to 40 patients with ABI or ND 2 weeks apart. Test–retest reliability was assessed using the intraclass correlation coefficient (ICC); clinical relevance of data was assessed through Pearson’s Correlation Coefficient with Montreal Cognitive Assessment (MoCA), the Community Integration Questionnaire – Revised (CIQ-R), and Hospital Anxiety and Depression Scale (HADS).

**Results:**

ICC values ranged from 0.86 to 0.93 for total and subscales. Significant correlations were found between MPAI-4-S-dig participation and CIQ-R Total, social integration and home integration and MoCA naming, MPAI-4-S-dig adjustment and CIQ-R Social integration, MPAI-4-S-dig Total and all subscale scores and HADS Anxiety score, MPAI-4-S-dig Total, abilities and participation and HADS Depression.

**Conclusion:**

The demonstrated reliability and clinical relevance of MPAI-4-S-dig for patients undergoing neurorehabilitation permits the implementation of digital data capture in patients with mild acquired cognitive impairment.

## LAY ABSTRACT

Brain injuries and neurological diseases such as stroke or Parkinson’s disease can cause many problems for those affected. To provide best possible rehabilitation and in order to study the consequences of the aforementioned conditions, self-report scales are commonly used to understand patients’ experiences. The Mayo-Portland Adaptability Inventory – fourth edition (MPAI-4) is an internationally well-established scale that exists in a self-report version. However, rehabilitation of brain injury is not equally available everywhere and internet-based versions must be evaluated so information is not lost on the way. In this study, we examined the measurement properties of a digital version of MPAI-4, MPAI-4-S-dig, and found that it gives reliable measurements over 2 time points. Patient responses were consistent with theoretical assumptions, indicating usefulness in a clinical context. This study demonstrates potential to use internet-based self-rating for persons with mild impaired cognitive functions.

The consequences of acquired brain injuries (ABIs) such as stroke or traumatic brain injuries (TBI) and neurological disorders (NDs) such as multiple sclerosis or Parkinson’s disease are multifaceted, causing diminished functional capacity in a range of areas (1).The Mayo Portland Adaptability Inventory – fourth edition (MPAI-4) is a clinical rating scale developed to measure global functioning following ABI, which has also found usage for ND (e.g., multiple sclerosis) (2). MPAI-4 is designed to be used in the formulation of rehabilitation outcomes and can be used by clinicians, close significant others, or as a self-assessment tool (3). Different raters have varying perspectives and biases. Clinicians tend towards objectivity, but may minimize subjective experiences, close significant others tend to overestimate patient difficulties, and patients typically overestimate the impact on abilities while underestimating effects of ABI on participation and adjustment, suggesting impaired self-awareness as a factor (4).

MPAI-4 has solid evidence for validity and reliability in its original design (5), provided in a pen-and-paper format. There is evidence suggesting equivalence between self-report scales investigating psychological well-being and psychiatric symptoms provided in a pen-and-paper format and similar instruments provided digitally (6). There are, however, studies suggesting differential neural processing of digital and analogue information (7). It is thus important not to assume equivalency but to evaluate internet-delivered self-report forms (8).

The use of digital self-report instruments has benefits in terms of optimizing caregiver resources, as well as for research, as they loosen reliance on time and place (9). They do, however, presuppose that patients are able to adequately rate and remember deficits, understand information presented to them, as well as self-monitor and interact with questionnaires. Patients with ABI commonly present with cognitive deficits, limitations in self-awareness, and emotional problems (10), which could potentially impair their capacity to adequately self-report their status (11,12). Type of injury, its localization, and mood have been proposed as mediating factors for patients’ ability to self-report accurately (13–15).

There is a need for more research on the implications of potential impairments to capacity for use of digital communication channels (16). It is thus important to evaluate online versions of self-report scales, as they form a mode of communication between patients, clinicians, and researchers. There is increasing evidence suggesting electronic modes of capturing patient-reported outcomes can be accurate and increase data quality and cost efficiency (17). MPAI-4 has been evaluated as a self-report instrument in the pen-and-paper format, showing similar levels of reliability to ratings by close significant others (18). However, there is a need to evaluate whether electronic data capture is feasible for patients experiencing cognitive deficits as a consequence of ABI or ND.

The present study has 2 primary objectives:

1. To investigate whether MPAI-4-S-dig can be used to provide clinically relevant and usable data in patients with ABI and ND.
2. To investigate whether the digital self-report version of MPAI-4 (MPAI-4-dig) is reliable for repeated measurements.

## METHODS

### Study setting

The patients were all enrolled by community neurorehabilitation teams in the Greater Stockholm area. Community neurorehabilitation teams are multiprofessional teams composed of physiotherapists, occupational therapists, speech and language therapists, and social workers. They conduct rehabilitation in the home and community for patients with ABIs and neurological disorders. To support the teams, neuropsychologists are stationed centrally at specialist rehabilitation hospitals and are available for consulting, regular supervision of team members, and neuropsychological assessment when needed. Rehabilitative efforts are conducted either in the patient’s home or at the community care centre. The patients are enlisted to the teams at varying stages, both transitioning from inpatient care and established in the home, presenting with different phases of chronic illness (19).

### Sample

Patients (*n* = 40) were between 18 and 83 years old and all fulfilled the inclusion criteria to the study, which were (*i*) that they were adults (18 years or older), (*ii*) presented with some cognitive sequelae along with motor impairments following ABI or ND, and (*iii*) showed mild to moderate cognitive deficits defined as a score on the Montreal Cognitive Assessment (MoCA) (20) at or above 21 out of 30. Patients with suspected Alzheimer’s disease were excluded. To be included, the participants should also have adequate visual-communicative and language abilities, access to a computer and internet connection, as well as a sufficiently stable social situation to undertake work consistently (i.e., homelessness would be an exclusion criterion). Patients presenting with comorbid conditions (i.e., substance abuse, severe and enduring mental illness) considered by therapists as impeding participation were also excluded. All participating patients received written and verbal information before signing informed consent forms. Demographic characteristics are presented in Table I.

**Table I:** Demographic characteristics of the participants (*n* = 40)

Characteristic	*n* (%)
Gender, *n* (%)	
Male	13 (32.5)
Female	27 (67.5)
Age range, mean (SD)	18–83, 52.1 (16.7)
Years of education	
≤ 9	4 (10.3)
≤ 12	15 (38.5)
≤ 15	20 (51.3)
Living alone or with partner	
Living with partner	26 (66.7)
Living alone	13 (33.3)
Work involvement	
100–75%	7 (18)
50%	9 (23.1)
25%	5 (12.8)
0%	18 (46.2)
Diagnosis	
Traumatic brain injury	4 (10.3)
Post-concussive syndrome	4 (10.3)
Stroke	12 (30.7)
Brain tumour	6 (15.4)
Postinfectious conditions	6 (15.4)
Neurological disorders	4 (10.3)
Other	3 (7.7)

Postinfectious conditions include meningitis and post-COVID condition, neurological disorders include Parkinson’s disease and multiple sclerosis.

### Study design: psychometric study

*Procedure*. The presented data were collected as a sub-study of a randomized controlled trial investigating the effects of internet-based cognitive rehabilitation for patients with mild to moderate cognitive deficits following ABI or ND (Clinical trials ID: NCT06973018). Eligible patients were enrolled to evaluate the reliability and validity of the digital version of MPAI-4 (MPAI-4-S-dig); 40 patients completed self-report measurements online 2 weeks apart. The participants also completed digital versions of the Community Integration Questionnaire (CIQ) and the Hospital and Anxiety Depression Scale on the national eHealth platform Stöd och Behandling (in English, “Support and treatment”) on 2 occasions, 2 weeks apart before the start of the intervention. As part of inclusion in the study patients underwent assessment with MoCA by a registered occupational therapist at baseline. Data included in this study were collected before commencement of rehabilitation.

*Measurements*. Three different measures were applied in digital format.

*1. Mayo-Portland Adaptability Inventory – fourth edition-Swedish digital version (MPAI-4-s-dig)* (3). In the present study, our research group developed a digital version of MPAI-4. The official translation of MPAI-4 into Swedish (21) was adapted.

The original scale consists of 29 questions covering 3 broad categories of what may be affected by cerebral injury: Functioning, Adaptation to illness, and Participation in society. Answers to questions from these 3 categories can then be calculated and a standardized score on each obtained. In addition to these categories, 6 additional questions regarding potential needs of patients undergoing assessment and rehabilitation are included in the original form. These incorporate alcohol and drug abuse, legal transgressions, psychotic symptoms, and potential comorbid conditions causing physical or cognitive impairment. The original 29 questions forming the standardized portion of the scale were maintained in the digital format; the 6 supplementary questions were therefore discarded, as they do not contribute to the subscales.

The original MPAI-4 was developed to capture difficulties and important outcomes when screening patients following acquired brain injuries. It is a scale designed to be used by professional staff, patients themselves, as well as close significant others to patients. Questions are based on examples and possible answers range from 0, indicating minimal or no issue, to 4, indicating a severe issue. Studies using classical test theory to examine reliability have found Cronbach’s alpha > 0.70. MPAI-4 has been validated in its differing iterations and in different patient groups (4).

2. *Montreal Cognitive Assessment (MoCA)* is a screening tool originally designed as an easily administrable way of capturing signs of mild cognitive impairment. Studies have found MoCA to be a sensitive and specific screening tool for cognitive impairment following other conditions, such as stroke, Parkinson’s disease, and heart failure (22). MoCA consists of brief tasks, adapted from established neuropsychological instruments measuring cognitive functions. The functions targeted in MoCA are short-term memory, executive function, language, visuospatial ability, attention and working memory, and orientation to time and place (27). MoCA is a 30-point scale: < 26 is the recommended cut-off point to detect mild cognitive impairment (23); < 22 points have been found to be an optimal cut-off point for detecting cognitive impairment in stroke populations (24), and was used in the study. However, it should be noted that reasons for utilizing a lower threshold for ABI patients include the prevalence of motor and speech impairments in the population concerned. Such impairments make test participation difficult without necessarily indicating cognitive deficits.

3. *The Community Integration Questionnaire – Revised (CIQ-R)* (25) is designed to capture community integration following traumatic brain injury. It is an 18-item scale examining the integration into home, social life, and productive activities. The scale exists as both a self-report measure and as a rating instrument for clinicians. Questions are rated from 0 to 2, indicating degree of participation in exemplified activities, with 2 indicating higher participation. Scores are presented as total and 4 subscales: home integration, social integration, productivity, and electronic social networking. Test–retest reliability for the pen-and-paper version was examined in an Australian sample during the development of the scale, where correlations between responses 2 weeks apart ranged between 0.66 and 0.94 for the different subscales (26). Significant but weak to moderate associations (–0.20 to –0.50) have been demonstrated between total and most subscales of MPAI-4 and CIQ, respectively in a population of military service members undergoing rehabilitation for traumatic brain injury (27)

4. *The Hospital Anxiety and Depression rating Scale (HADS)* is developed to capture symptoms of depressive and anxious symptomatology among patients affected by mental illness (28). It was conceived as a self-report scale from the outset, to be used in non-psychiatric settings. It is a 14-item scale, where questions are rated from 0 to 3, where 0 indicates no problem. Scores are provided in 2 subscales, depression and anxiety, respectively. A cut-off score of 11 is considered a high probability of a clinical depression or anxiety (28). A Swedish study examining internet-delivered HADS (29) found test–retest correlations at *r* = 0.84 for HADS-Depression and *r* = 0.71 for HADS-Anxiety.

### Statistical methods

The data were checked for normality distribution. Means, standard deviation, and range for demographic variables, means, and standard deviation of the 3 subscales of the MPAI-4, subscales of the CIQ, and subscales of MoCA were calculated. Test–retest reliability was calculated using intraclass correlation (30). Construct validity is calculated with Pearson product moment correlation. SPSS version 27 was used for all analyses (IBM Corp, Armonk, NY, USA). *P*-values less than or equal to 0.05 were considered statistically significant.

### Ethics

This study was approved by the Swedish ethical review authority, diary no. 2020-03079.

## RESULTS

A total of 40 patients completed the MPAI-4-S-dig on 2 occasions, 2 weeks apart. Patients represent an aetiologically heterogenous population with an age span from 18 to 83, affected by differing conditions as described in Table I.

Reliability measurements for MPAI-4-S-dig are described in Table II. Intraclass correlations (ICC) indicate acceptable reliability for Total, and all the subscales. All mean values were within the average range (T = 40–60) for patients participating in brain injury rehabilitation (3).

**Table II:** Mayo-Portland Adaptability Inventory – fourth edition Swedish Digital version (MPAI-4-s-dig): means at measurements 2 weeks apart (*n* = 40)

MPAI-4-s-dig Subscale score	Test T1 Mean (SD)	Test T2 Mean (SD)	ICC	95% CI
Total	28.2 (12.6)	26.36 (12.1)	0.92	0.85–0.96
Participation	9.2 (5.3)	8.4 (5.7)	0.93	0.87–0.96
Abilities	13.5 (6.5)	12.7 (6.3)	0.86	0.78–0.94
Adjustment	11.1 (5.5)	9.4 (4.9)	0.92	0.86–0.96

Intraclass correlations (ICC) and confidence interval (CI) are presented.

Patients’ results on MoCA are described in detail in Table III; the mean total score is above the cut-off point for detecting mild cognitive impairment. MoCA scores were evaluated for normality using a Kolmogorov–Smirnov test and were found not to be distributed normally; median and interquartile range (IQR) are thus additionally presented.

**Table III:** Montreal Cognitive Assessment (MoCA) scores at baseline

MoCA scores	Mean (SD), median, IQR
Total	26.9 (2.3) 27, 25–29
Visuospatial/Executive	4.6 (0.73), 5, 5–5
Naming	2.9 (.22) 3, 3–3
Attention	5.7 (.75) 6, 6–6
Language	2.5 (.81) 3, 2–3
Abstraction	1.7 (.42) 2, 2–2
Delayed recall	3.4 (1.3) 4, 2–5
Orientation	5.8 (.44) 6, 6–6

Mean, median, and IQR are presented for the total value.

Reliability measures for CIQ-R are described in detail in Table IV. ICC values were found to be acceptable for the total scale and all subscales. All results, total and subscales were within the average range according to Australian norms (25).

**Table IV:** Community Integration Questionnaire – Revised (CIQ-R): means at measurements 2 weeks apart

CIQ-R score	Test T1 Mean (SD)	Test T2 Mean (SD)	ICC	95% CI
Total	20.0 (5.9)	19.7 (5.9)	0.93	0.86–0.96
Home integration	7.3 (3.0)	7.0 (2.9)	0.96	0.93–0.98
Social integration	6.4 (1.7)	6.3 (1.6)	0.74	0.50–0.86
Productivity	3.6 (2.3)	3.4 (2.4)	0.71	0.45–0.84
Electronic networking	2.8 (1.9)	3.2 (1.7)	0.86	0.73–0.92

Intraclass correlations (ICC) and 95% confidence interval (CI) are also presented.

Test–retest reliability for HADS is described in Table V. It was found to be acceptable for total score as well as the anxiety and depression subscales.

**Table V:** Hospital Anxiety and Depression Scale (HADS): means, ICC, and confidence interval at 2 weeks apart

HADS Subscale	Test T1 Mean (SD)	Test T2 Mean (SD)	ICC	95% CI
Anxiety	6.4 (3.63)	6.16 (3.92)	0.93	0.87–0.96
Depression	7.60 (2.57)	7.05 (2.64)	0.70	0.42–0.84
Total	14.20 (4.71)	13.48 (5.33)	0.89	0.79–0.94

Pearson’s test revealed an absence of significant correlations between most MPAI-4-S-dig measurements and MoCA, with the exception of the MPAI Participation subscale and MoCA Naming subtest as is demonstrated in Table VI.

**Table VI:** Pearson correlation coefficients for Mayo-Portland Adaptability Inventory – fourth edition Swedish Digital version (MPAI-4-S-dig) and Montreal Cognitive Assessment (MoCA)

Item	MoCA VSE	MoCA N	MoCA A	MoCA L	MoCA Ab	MoCA DR	MoCA O	MoCA Total
MPAI-4-S-dig Total	–0.06	–0.23	0.24	–0.10	–0.16	0.04	0.06	–0.12
MPAI-4-S-dig Adjustment	0.22	–0.18	–0.27	–0.13	–0.23	0.03	0.06	–0.08
MPAI-4-S-dig Abilities	–0.16	–0.05	–0.05	–0.01	–0.07	–0.01	0.03	–0.06
MPAI-4-S-dig Participation	–0.02	–0.34*	–0.23	–0.26	–0.11	0.01	0.05	–0.17

VSE: Visuospatial/executive; N: Naming; A: Attention; L: Language; Ab: Abstraction; DR: Delayed recall; O: Orientation.

* *p* = < 0.05.

Results of Pearson’s correlation revealed that the MPAI subscale Participation correlates negatively with CIQ-R Total score, Social integration and home integration, which is displayed in Table VII.

**Table VII:** Pearson’s correlation coefficient Mayo-Portland Adaptability Inventory – fourth edition Swedish Digital version (MPAI-4-S-dig) and Community Integration Questionnaire – Revised (CIQ-R)

Item	CIQ-R Total	CIQ-R SI	CIQ-R HI	CIQ-R P	CIQ-R E
MPAI-4-S-dig Total	–0.25	–0.29	–0.12	–0.16	–0.16
MPAI-4-S-dig Participation	–0.49**	–0.52**	–0.32*	–0.24	–0.24
MPAI-4-S-dig Abilities	0.005	–0.03	–0.002	0.04	–0.04
MPAI-4-S-dig Adjustment	–0.29	–0.32*	–0.13	–0.16	–0.16

SI: Social integration; HI: Home integration; P: Productivity; E: Electronic social networking.

* *p* = < 0.05.

** *p* = < 0.01.

Moderate to weak associations using Pearson’s correlation were found between all measures of MPAI-4-S-dig and self-reported anxiety (Table VIII) and weak associations were found between depressive symptomatology and MPAI-4-S-dig total, abilities and participation as shown in Table VIII. When utilizing HADS total score, moderate associations were found between all MPAI-4-S-dig measures.

**Table VIII:** Pearson’s correlation coefficients HADS and MPAI-4-S-dig

Item	MPAI-4-S-dig Total	MPAI-4-S-dig Adjustment	MPAI-4-S-dig Abilities	MPAI-4-S-dig Participation
HADS Anxiety	0.56**	0.64*	0.38*	0.40**
HADS Depression	0.32*	0.27	0.32*	0.34*
HADS Total	0.60**	0.60**	0.45**	0.46**

* *p* = < 0.05.

** *p* = < 0.01.

## DISCUSSION

The aim of this study was to investigate the feasibility of using a digital version of MPAI-4 by examining the test–retest reliability and validity of the digital version of MPAI-4-S-dig. Self-reported symptoms following brain injury were collected on 2 occasions. Patients formed a heterogenous group, diagnosed with a variety of illnesses and injuries affecting the brain and central nervous system and undergoing rehabilitation in a community setting. The data collection was a part of a randomized controlled trial examining internet-delivered cognitive rehabilitation; 43 patients were invited to complete the self-report measurements described. The vast majority of invited patients successfully completed the digital self-report form, with few (7%) dropouts. Dropouts represent patients who discontinued participation in the rehabilitation programme overall, rather than dropouts specifically from the psychometric portion that is the focus of this study. Patients also indicated that they experienced difficulties in abilities, adjustment, and participation in everyday life in the MPAI-4-S-dig at a level typical of patients admitted to brain injury rehabilitation clinics, suggesting representativeness of the sample.

Consistency of measurement was found between the 2 measurement points where ICC values were ranging from 0.86 to 0.93, indicating stability of measurement for the digital version. This result indicates that a group of patients diverse in age and aetiology provide reliable symptom reports in a digital format. Similar findings, with ICC values above 0.70, have been found in studies investigating pen-and-paper versions (2).

There is a fundamental discrepancy between results from a cognitive screening test (MoCA), which indicated relatively intact cognitive functioning, and reports on everyday life functioning as captured by the different subscales of MPAI-4. The absence of correlation between impairments as reported on the MPAI-4-S-dig and aspects of MoCA (with the exception of a weak correlation between Participation and Naming) is indicative of the importance of linguistic communication abilities to participation in everyday life.

However, the results of the other correlations are consistent with previous studies indicating separation between objective measures of cognitive function and self-reported deficits (31). This discrepancy is in line with earlier finding from Malec et al. (4) showing a tendency for patients to overestimate deficits in the pen-and-paper versions and underlines the need to collect information on patient needs and functioning from multiple perspectives. In this study, this was done through the use of several different measurement tools.

Considering validity, consistency was also found between reports on MPAI-4-S-dig and associated measures on the CIQ-R. This is consistent with theoretical assumptions suggesting shared constructs, i.e., measurements of participation in the MPAI-4-S-dig correlate with measures of participation in the CIQ-R. In this study, fewer associations were found between CIQ-R and MPAI-4-S-dig than in a study investigating associations between CIQ and MPAI-4 among military service members affected by traumatic brain injury (18). However, the population included in this study was more heterogeneous in background, age, and aetiology than in that particular study, making conclusive comparisons difficult.

In this study significant associations were found between anxiety on HADS, and all subscales of MPAI-4-S-dig, and 3 out of the 4 measurements of MPAI-4-S-dig and HADS depression. Scores for anxiety indicate that patients experience symptoms bordering, but not crossing, the threshold to mild clinically relevant anxiety. Average depression rating indicates mild depressive symptomatology (32). Taken together these findings would suggest relatively low levels of psychological distress among patients participating in the study. However, there are studies suggesting the 2 factors, anxiety and depression, assumed by HADS do not fit as well as a single factor indicating general distress for brain injury patients (33). When correlating the total score for HADS, moderate associations were found between general distress and all MPAI-4-S-dig measures. Previous studies have identified mood as a moderating factor for self-reported deficits following neurological disorders and brain injuries (34), supporting the clinical relevance of these findings to brain injury-specific outcomes regardless of whether patients fulfil the original criteria of HADS.

### Study limitations

The present study was done without a pen-and-paper comparison group, which may be considered a potential weakness. However, comparing with earlier published studies might indicate comparability of digitally obtained data. Furthermore, the focus in this study was to explore the feasibility and usability of the digital instrument itself. Examining the equivalence between digital and pen-and-paper administration of MPAI-4 may be considered a viable future study given the results we have obtained.

Patients included in the study had, as mentioned, high scores obtained on MoCA. The use of MoCA has the advantage of ease of use and it is a widely spread instrument. However, MoCA is a screening instrument. An admittedly time-consuming full-scale neuropsychological assessment requiring specialized training and experience would likely capture subtle deficits that MoCA does not. Patients included in the study represent only a relatively well-functioning segment of the outpatient population of ABI and ND patients who could potentially have greater ease in using digital instruments than more severely affected patients. This segment, however, is relatively large (35).

The possibility that the patients who chose to participate in the study are misrepresenting the patient population in other ways, such as having a more positive attitude towards digital technology in general, cannot be entirely dismissed. Inclusion in this study is based on suitability to participate in a rehabilitation programme, not specific to evaluation of online self-report. The heterogenous nature of the participants suggests representativeness; however, the lack of data on patients who were excluded by therapists from entering the programme is an obstacle to decisive conclusions.

Patients completed self-report questionnaires in their home environment, rather than in a hospital or care facility setting. Although the lack of control over contextual factors could be considered a limitation to the study, all patients successfully completed the digital self-assessment at least once, with a low attrition rate for completing it a second time. Given the controlled nature of healthcare settings, a high compliance rate in a natural setting supports the feasibility of digital self-assessment.

The sample size for the study was relatively small, and it is possible that in a larger sample additional factors could have been elucidated.

### Clinical conclusion

MPAI-4 in its paper-and pencil format is a time- and resource-demanding tool. Its demonstrated reliability and validity in a digital format indicate the possibility of implementation of time-saving digitalisation for patients with mild to moderate cognitive deficits, who form a large patient population. On a general level this study furthermore supports digital data capture when providing treatment or treatment planning for patients with deficits following nervous system disease or injury associated with potential cognitive symptoms. The results also further knowledge on how self-reported data relate to each other, elucidating seemingly contradictory results.
